# Food elimination diet is a viable alternative therapy for eosinophilic esophagitis responsive to proton pump inhibitors

**DOI:** 10.1186/s12876-023-02703-9

**Published:** 2023-03-09

**Authors:** Twan Sia, Evan Cunningham, Megan Miller, Rebecca Nitschelm, Riki Tanaka, Taylor Epstein, Kendall Garrett, Amy Huang, Daniel Pak, Ally Scheve, John Leung

**Affiliations:** Boston Specialists, 65 Harrison Ave Suite #201, Boston, MA 02111 USA

**Keywords:** PPI, FED, Eosinophils, EoE

## Abstract

**Background:**

First-line treatment of eosinophilic esophagitis (EoE) includes monotherapy with proton-pump inhibitors (PPIs), food elimination diet (FED), or topical corticosteroids. Current guidelines suggest patients with EoE should continue any responsive first-line monotherapies. However, the efficacy of FED monotherapy in patients with EoE responsive to PPI monotherapy has not been well studied. Our study aimed to investigate how attempting FED monotherapy after experiencing remission of EoE after PPI monotherapy influenced long-term EoE management.

**Methods:**

We retrospectively identified patients with EoE responsive to PPI monotherapy who trialed FED monotherapy. We then employed a mixed method approach to a prospective cohort. Selected patients were observed long term for quantitative outcomes, while qualitative results were obtained from patient surveys regarding their perspectives on the trial of FED monotherapy.

**Results:**

We identified 22 patients who trialed FED monotherapy after experiencing remission of EoE following PPI monotherapy. Of these 22 patients, 13 had remission of EoE with FED monotherapy, while 9 had re-activation of EoE. Out of 22 patients, 15 were enrolled in a cohort for observation. No exacerbations of EoE occurred while on maintenance treatment. Most patients stated that they would recommend this process to others with EoE (93.33%) and that trial of FED monotherapy helped them identify a treatment plan that aligned with their lifestyle (80%).

**Conclusion:**

Our work shows that FED monotherapy can be an effective alternative for patients with EoE responsive to PPI monotherapy that may improve patient quality of life, suggesting alternative treatment options should be considered for monotherapy-responsive EoE.

**Supplementary Information:**

The online version contains supplementary material available at 10.1186/s12876-023-02703-9.

## Background

Eosinophilic esophagitis (EoE) is a chronic inflammatory disease of the esophagus. In adults, EoE commonly presents with dysphagia to solid foods, food impaction, chest pain, and heartburn. Diagnosis of EoE is based on histologic findings of ≥ 15 eosinophils per high-power field (eos/hpf) upon esophageal biopsies in patients with esophageal dysfunction [1, 2].

Current treatment algorithms recommend proton pump inhibitors (PPIs), food elimination diets (FEDs), and topical corticosteroids as first-line therapies for EoE [3, 4]. These treatments have been shown to be highly effective in inducing histologic remission of EoE, as traditionally defined by < 15 eos/hpf, after several weeks of treatment [5–7]. Guidelines suggest that if a patient’s EoE is not histologically responsive to a given first-line therapy, alternative first-line treatments should be attempted followed by repeat esophagogastroduodenoscopy (EGD) for histologic re-evaluation until each therapeutic option has been exhausted [3, 4]. Because current guidelines are focused on inducing histologic remission following a course of monotherapy, there is a paucity of research on the long-term management of and clinical decision making in treating EoE [4, 8].

From our clinical experience with treating EoE patients, once patients have identified a monotherapy that their EoE is responsive to, they may be interested in switching to a monotherapy that may better suit their lifestyle. However, the trial of first-line therapies in patients whose EoE has been shown to be responsive to a different treatment has not been well studied [3, 4], which can be at least partly attributed to the resource intensive nature of evaluating treatment success in EoE [9, 10].

While the efficacy of FED is well studied in patients with EoE unresponsive to PPI monotherapy [6], FED is rarely examined in patients with EoE responsive to PPI monotherapy (EoE^PPI+^). A previous case series reported 5 patients whose EoE was responsive to both PPI monotherapy and FED monotherapy (EoE^PPI+, FED+^) [11]. Furthermore, a recent retrospective study showed that 6 patients out of 9 adult EoE^PPI+^ patients were also responsive to FED monotherapy [12]. However, these studies have a relatively small number of patients. Furthermore, they do not describe how the trial of FED monotherapy in EoE^PPI+^ patients can influence the management of their EoE.

Our study had the following aims: 1) Corroborate previous findings by identifying the proportion of EoE^PPI+^ who are also responsive to FED monotherapy in a larger group of patients; 2) Report long-term outcomes of these patients, such as complications or health-care utilization; and 3) Describe EoE^PPI+^ patient perspectives of FED monotherapy trial, given the greater burden associated with trialing different EoE treatments [9, 10].

## Methods

### Study design, patients, and measures

We conducted a mixed methods study in two phases. Phase 1 was a retrospective study aimed at identifying the efficacy of FED monotherapy in EoE^PPI+^ patients. Phase 2 was a prospective cohort study that investigated patient outcomes after trialing both PPI monotherapy and FED monotherapy using both quantitative and qualitative outcomes. This study has received Institutional Review Board approval.

In Phase 1, we performed a chart review where EoE^PPI+^ patients who trialed FED monotherapy were identified using the International Classification of Diseases, Tenth Revision (ICD-10), code K20.0 (eosinophilic esophagitis) at a single center from January 2013 until September 2021. From 405 patients identified with the ICD-10 code K20.0, patients were included for chart review and subsequent analysis if they met the following criteria: 1) Diagnosis of EoE was histologically confirmed while not on any treatments for EoE, 2) Achievement of histologic remission of EoE while on at least 8 weeks of PPI monotherapy, and 3) Had repeat esophageal biopsies after trialing FED monotherapy for at least 8 weeks. In our study, histologic diagnosis of EoE was defined by ≥ 15 eos/hpf in esophageal biopsies taken during EGDs. Histologic remission of EoE was our primary endpoint and was defined by ≤ 10 eos/hpf in proximal, middle, and distal esophageal biopsies from EGDs after at least 8 weeks of respective monotherapy treatment. Histoclinical features such as symptoms and peak eosinophil counts were extracted from the electronic medical record. Symptoms were recorded as binary outcomes. Endoscopic reference scores (EREFS) were unavailable for many of the patients, so they were not included in our analysis.

During Phase 2 of our study, patients were enrolled into a prospective cohort on a voluntary basis until September 2021. EoE^PPI+, FED−^ patients resumed PPI monotherapy, while EoE^PPI+, FED+^ patients could revert to PPI monotherapy, continue FED monotherapy, or switch to FED monotherapy with PPI on an as needed basis. The exploratory option of FED monotherapy with PPI on an as needed basis was offered because these patients were responsive to both PPI monotherapy and FED monotherapy. Therefore, it was plausible that their EoE would remain in histologic remission with this regimen. We measured patient health-care utilization during this follow-up period by recording the number of patients with food impactions requiring urgent EGD and the number of patients who suffered from symptom exacerbation requiring urgent follow-up visit while on their maintenance treatment plan. We also recorded the number of patients that had repeat EGDs for routine monitoring of histologic recurrence of EoE while on maintenance treatment plan, and the number of patients who underwent repeat EGDs for histologic evaluation after trialing other treatment plans.

### Survey development and administration

In addition to quantitative endpoints, we obtained qualitative data through surveys investigating the patient’s perspective on the quality of care they received and their reasoning for trialing FED monotherapy even though they achieved remission of EoE while on PPI monotherapy. EoE^PPI+^ patients that trialed FED monotherapy answered a brief three-item survey, and EoE^PPI+, FED+^ patients answered an additional three-item survey. All questions were in multiple-choice format. The questionnaire design was developed by a clinician with EoE expertise (J.L.). Specific questions were designed with the goal of understanding patient motivations and quality of life following trial of PPI monotherapy and FED monotherapy for the management of EoE. All survey questions are listed in Tables 4 and 5. The survey was administered at the conclusion of Phase 2 via telephone interviews.

### Analysis

We analyzed patient demographics, clinical characteristics, and survey responses using descriptive statistics. For paired comparisons of histologic data, we used Wilcoxon signed-rank tests.

## Results

### Participants

**Fig. 1:**
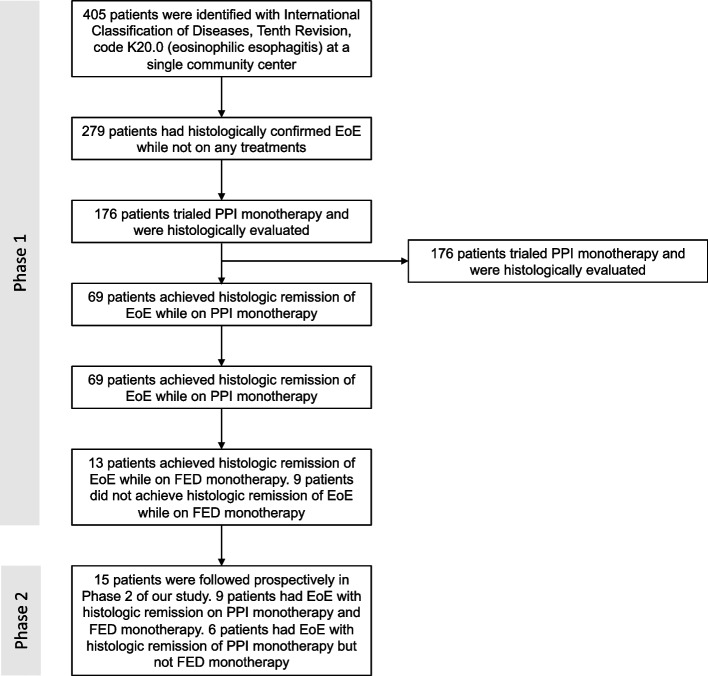
Flowchart of patients included in our retrospective cohort based on our inclusion criteria and in our prospective cohort based on voluntary enrollment

We identified 405 EoE patients using the ICD-10 code K20.0 for EoE. From 405 patients, 126 patients were excluded as they were diagnosed and started on a treatment plan elsewhere. From the remaining 279 patients, 176 patients had trialed PPI monotherapy, of which 107 patients had EoE that was not histologically responsive to PPI monotherapy, and 69 patients had EoE^PPI+^. Out of these 69 patients, 22 patients trialed FED monotherapy after cessation of PPI monotherapy and were therefore entered into our retrospective cohort (Fig. 1). The median age of these 22 patients was 34 years (IQR 29.2–39.7, Table 1), and 13 were male (59.09%; Table 1, Table S1).

**Table 1:** Demographics of patients with EoE that achieved histologic remission with PPI monotherapy in each phase of our study

	**Phase 1**		**Phase 2**	
**Characteristics**	**Patients with histologic remission of EoE on PPI monotherapy and trialed FED monotherapy (*n* = 22)**	**Patients with histologic remission of EoE on PPI monotherapy and FED monotherapy (*n* = 13)**	**Patients with histologic remission of EoE on PPI monotherapy and trialed FED monotherapy (*n* = 15)**	**Patients with istologic remission of EoE on PPI monotherapy and FED monotherapy (*n* = 9)**
Age, median (IQR)	34 (29.2–39.7)	37.9 (29.5–44)	33.25 (29.3–46.3)	38.4 (31.3–52.8)
Male, n (%)	13 (59.09%)	8 (61.54%)	10 (66.67%)	6 (66.67%)
Atopic comorbidity, n (%)				
Any atopic condition	11 (50%)	6 (46.15%)	7 (46.67%)	4 (44.44%)
Atopic dermatitis	0 (0%)	0 (0%)	0 (0%)	0 (0%)
Asthma	3 (13.64%)	1 (7.69%)	2 (13.33%)	1 (11.11%)
Allergic rhinitis	8 (36.36%)	4 (30.77%)	4 (26.67%)	2 (22.22%)
Food allergy	6 (27.27%)	4 (30.77%)	3 (20%)	2 (22.22%)
PPI monotherapy, n (%)				
Omeprazole 40 mg twice daily	14 (63.64%)	7 (53.85%)	10 (66.67%)	5 (55.56%)
Omeprazole 40 mg once daily	7 (31.82%)	5 (38.46%)	4 (26.67%)	3 (33.33%)
Omeprazole 20 mg twice daily	1 (4.55%)	1 (7.69%)	1 (6.67%)	1 (11.11%)
FED monotherapy, n (%)				
Dairy, wheat, soy, egg, nut FED	1 (4.55%)	0 (0%)	0 (0%)	0 (0%)
4FED	1 (4.55%)	1 (7.69%)	0 (0%)	0 (0%)
Dairy, wheat, soy FED	1 (4.55%)	0 (0%)	1 (6.67%)	0 (0%)
2FED	15 (68.18%)	8 (61.54%)	11 (73.33%)	6 (66.67%)
Dairy FED	4 (18.18%)	4 (30.77%)	3 (20%)	3 (33.33%)

*2FED* Two-food elimination diet (dairy and wheat food elimination diet), *4FED* Four-food elimination diet (dairy, wheat, soy, egg food elimination diet), *EoE* Eosinophilic esophagitis, *FED* Food elimination diet, *IQR* Interquartile range, *PPI* Proton pump inhibitor

### Proportion of patients with EoE responsive to PPI monotherapy that was also responsive to FED monotherapy

**Fig. 2:**
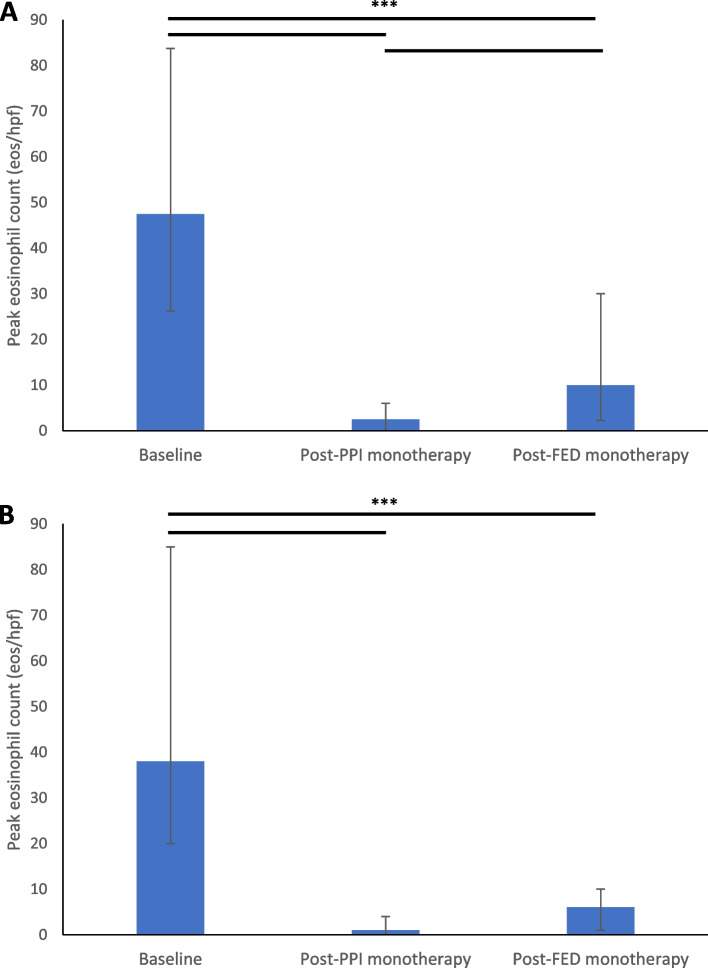
Comparison of median peak eosinophils per high-power field in baseline, post-PPI monotherapy, and post-FED monotherapy in (**A**) all 22 patients with EoE responsive to PPI monotherapy who trialed FED monotherapy in the retrospective phase of our study, and (**B**) the 13 patients who had EoE that was histologically responsive to PPI monotherapy and FED monotherapy. **A** Peak eosinophils per high-power field in baseline (median 47.5, IQR 26.25–83.75) versus post-PPI monotherapy (median 2.5, IQR 0–6), post-FED monotherapy (median 10, IQR 2.25–30). **B** Peak eosinophils per high-power field in baseline (median 38, IQR 20–85) versus post-PPI monotherapy (median 1, IQR 0–4), post-FED monotherapy (median 6, IQR 1–10). Error bars represent the interquartile range. Paired comparisons were made using the Wilcoxon Signed Rank Test. *** indicates *p* < 0.001

In Phase 1 of our study, all 22 patients were diagnosed with histologically confirmed EoE (median peak eosinophil count 47.5 eos/hpf, IQR 26.25–83.75; Table 2). All patients were symptomatic with most patients suffering from dysphagia (81.82%, Table 2). All 22 patients were histologically responsive to PPI monotherapy, although dosages and frequencies varied between patients. The most popular PPI monotherapy was omeprazole 40 mg twice daily (63.64%, Table 1). While on PPI monotherapy, most patients were asymptomatic (63.64%, Table 2). However, reported symptoms included dysphagia (22.73%, Table 2), heartburn (13.64%, Table 2), vomiting (4.55%, Table 2), abdominal pain (4.55%, Table 2), and regurgitation (4.55%, Table 2). EGD while patients were on PPI monotherapy revealed a median peak eosinophil count of 2.5 eos/hpf (IQR 0–6, Table 2), significantly less than at baseline (median 47.5, IQR 26.25–83.75; Fig. 2, Table S1).

**Table 2:** Histoclinical characteristics of patients that achieved histologic remission of EoE on PPI monotherapy during baseline, PPI monotherapy trial, and FED monotherapy trial

Characteristics	Baseline, all patients (*n* = 22)	PPI monotherapy, all patients (*n* = 22)	FED monotherapy, all patients (*n* = 22)	FED monotherapy, patients histologicly responsive to FED monotherapy (*n* = 13)
Peak eos/hpf, median (IQR)	47.5(26.25–83.75)	2.5 (0–6)	10 (2.25–30)	6 (1–10)
Symptoms, n (%)				
Dysphagia	18 (81.82%)	5 (22.73%)	4 (18.18%)	2 (15.38%)
Food Impaction	3 (13.64%)	0 (0%)	0 (0%)	0 (0%)
Heartburn	9 (40.91%)	3 (13.64%)	5 (22.73%)	4 (30.77%)
Chest pain	4 (18.18%)	0 (0%)	0 (0%)	0 (0%)
Vomiting	2 (9.09%)	1 (4.55%)	0 (0%)	0 (0%)
Abdominal pain	3 (13.64%)	1 (4.55%)	0 (0%)	0 (0%)
Regurgitation	1 (4.55%)	1 (4.55%)	0 (0%)	0 (0%)
Asymptomatic	0 (0%)	14 (63.64%)	15 (68.18%)	8 (61.54%)

*eos/hpf* eosinophils per high-power field, *FED* Food elimination diet, *IQR* Interquartile range, *PPI* Proton pump inhibitor

All 22 patients trialed FED monotherapy after cessation of PPI monotherapy. Patients were on a variety of FEDs, with the most popular being dairy and wheat FED (two-food elimination diet, 2FED; 68.18%; Table 1). Most patients on FED monotherapy reported being asymptomatic (68.18%, Table 2). Symptomatic patients reported heartburn (22.73%, Table 2) and dysphagia (18.18%, Table 2). While on FED monotherapy, these 22 patients had a median peak eosinophil count of 10 eos/hpf (IQR 2.25–30; Table 2, Table S1).

Out of 22 EoE^PPI+^ patients who trialed FED monotherapy, 13 patients (59.09%; Fig. 1) were determined to have EoE^PPI+, FED+^, while 9 patients (40.91%; Fig. 1) did not achieve histologic remission of EoE with FED monotherapy (EoE with histologic remission to PPI monotherapy but not FED monotherapy, EoE^PPI+, FED−^). Thirteen EoE^PPI+, FED+^ patients had a median peak eosinophil count of 6 eos/hpf (IQR 1–10, Table 2) while on FED monotherapy, which was significantly less than they had at baseline (median 38, IQR 20–85; Fig. 2, Table S1).

### Observations within the prospective cohort

Following trial of FED monotherapy, 15 patients out of 22 total patients were voluntarily enrolled in a prospective cohort for observation in Phase 2 of our study. Of these 15 participants, 9 were EoE^PPI+, FED+^ and 6 were EoE^PPI+, FED−^ (Fig. 1). During this observation period, patients with EoE^PPI+, FED−^ resumed PPI monotherapy, while EoE^PPI+, FED+^ patients were given the option to revert to PPI monotherapy, continue FED monotherapy, or start FED monotherapy with PPI on an as needed basis. Median follow up duration for EoE^PPI+, FED+^ patients was 2.25 years (IQR 1.51–2.48, Table 3), and median follow up duration for EoE^PPI+, FED−^ patients was 1.08 years (IQR 0.73–2.38; Table 3, Table S2).

**Table 3:** Incidents during cohort observation period

Recorded Metrics	Patients with histologic remission of EoE on PPI monotherapy and FED monotherapy (*n* = 9)	Patients with histologic remission of EoE on PPI but not FED monotherapy (*n* = 6)
Length of follow up, median yr (IQR)	2.25 (1.51–2.48)	1.08 (0.73–2.38)
Patients with food impactions requiring urgent EGD, n (%)	0 (0%)	0 (0%)
Patients with symptom exacerbation requiring urgent follow up visit, n (%)	0 (0%)	0 (0%)
Pts who underwent repeat EGD while on maintenance treatment plan, n (%)	1 (11.11%)	2 (33.33%)
Patients with histologic reactivation of EoE while on maintenance treatment plan, n (%)	0 (0%)	0 (0%)
Patients who underwent repeat EGD to trial other treatment plans, n (%)	4 (44.44%)	4 (66.67%)

*EGD* Esophagogastroduodenoscopy, *EoE* Eosinophilic esophagitis, *FED* Food elimination diet, *IQR* Interquartile range, *PPI* Proton pump inhibitor

During the observation period, we recorded patient health-care utilization due to exacerbation while on maintenance treatment or trial of other treatment plans for EoE. Health-care utilization was similar between EoE^PPI+, FED+^ and EoE^PPI+, FED−^ patients. No patients had food impactions warranting urgent EGD or symptom exacerbation requiring urgent follow up visit (Table 3). One patient with EoE^PPI+, FED+^ (11.11%, Table 3) and two EoE^PPI+, FED−^ patients (33.33%, Table 3) underwent repeat EGD while on maintenance treatment plan for histologic re-evaluation. None of these patients had histologic reactivation of EoE while on maintenance treatment plan (Table 3). Four EoE^PPI+, FED+^ patients (44.44%, Table 3) and four EoE^PPI+, FED−^ patients (66.67%; Table 3, Table S2) had a repeat EGD for histologic evaluation of other treatment plans. These EGDs showed histologically reactivated EoE, so patients restarted their maintenance treatment plan following these empirical trials.

### Qualitative results from prospective cohort

After the observation period, all 15 patients in the prospective cohort answered a three-item survey. When asked about why they pursued trial of FED monotherapy after knowing that their EoE was responsive to PPI therapy, a majority of patients (60%, Table 4) were concerned about long-term medication usage. Other patients cited that they suspected having side effects due to PPI monotherapy (13.33%), wanted to discover their food triggers (20%), or wanted options for future treatment (6.67%). When considering a FED monotherapy trial after having histologic remission with PPI monotherapy, a majority of patients answered that they would recommend this process for someone else with EoE (93.33%) and that they would personally go through this process again (80%).

**Table 4:** Patient opinions on the trial of FED monotherapy after achieving histologic remission of EoE with PPI monotherapy

Survey responses	Patients with histologic remission of EoE on PPI monotherapy and FED monotherapy (*n* = 9)	Patients with histologic remission of EoE on PPI monotherapy but not FED monotherapy (*n* = 6)	All patients (*n* = 15)
*Question: After finding out that your EoE was responsive to PPI therapy, why did you choose to undergo a FED and repeat EGD?*			
Concerned about long-term medication usage	5 (55.56%)	4 (66.67%)	9 (60%)
Suspected side effects of PPI	1 (11.11%)	1 (16.67%)	2 (13.33%)
Wanted to discover food triggers	2 (22.22%)	1 (16.67%)	3 (20%)
Wanted options for future treatment	1 (11.11%)	0 (0%)	1 (6.67%)
*Question: Would you recommend this process for someone else with EoE?*			
Yes	8 (88.89%)	6 (100%)	14 (93.33%)
*Question: Would you personally go through this process again?*			
Yes	8 (88.89%)	4 (66.67%)	12 (80%)

*EGD* Esophagogastroduodenoscopy, *EoE* Eosinophilic esophagitis, *FED* Food elimination diet, *PPI* Proton pump inhibitor

The 9 patients who had EoE^PPI+, FED+^ answered an additional survey. Given that they had histologic remission to PPI monotherapy and FED monotherapy, patients had options for their maintenance treatment plan. A majority of patients decided to continue FED monotherapy (55.56%, Table 5), some chose to switch to FED monotherapy with PPI on an as needed basis (33.33%, Table 5), and others reverted to PPI monotherapy (11.11%, Table 5). When asked why they were following their particular maintenance treatment plan over other options, 66.67% answered that their treatment plan was more sustainable for them, and 33.33% answered they perceived that their current treatment plan had better symptom benefits (Table 5). A majority of patients also strongly agreed (55.56%, Table 5) that undergoing a FED monotherapy trial after knowing that PPI monotherapy induced histologic remission of their EoE had increased their overall quality of life and helped them identify a treatment plan that aligned with their lifestyle and beliefs.

**Table 5:** Maintenance therapy in patients with EoE that achieved histologic remission with PPI monotherapy and FED monotherapy

Survey responses	Patients with histologic remission of EoE on PPI monotherapy and FED monotherapy (*n* = 9)
*Question: What treatment plan do you primarily follow?*	
PPI monotherapy	1 (11.11%)
FED monotherapy	5 (55.56%)
FED monotherapy with PPI on as needed basis	3 (33.33%)
*Question: Why are you following this treatment plan?*	
Current treatment plan is sustainable	6 (66.67%)
Perceived symptom benefits of current treatment plan	3 (33.33%)
*Question: Do you believe that this process has increased your overall quality of life with your EoE and helped you to identify a treatment plan that aligned with your lifestyle and beliefs?*	
Strongly agree	5 (55.56%)
Agree	2 (22.22%)
Neutral	2 (22.22%)
Disagree	0 (0%)
Strongly disagree	0 (0%)

*EoE* Eosinophilic esophagitis, *FED* Food elimination diet, *PPI* Proton pump inhibitor

## Discussion

Although there are numerous effective first-line therapies for inducing histologic remission of EoE, current guidelines lack recommendations for patients after they achieve histologic control of their EoE. In particular, there are relatively few studies on maintenance treatment of EoE [4, 8] and few recommendations for EoE^PPI+^ patients other than to continue PPI monotherapy [3, 4]. In this study, we investigated FED monotherapy trial in EoE^PPI+^ patients.

Previous work identified 5 EoE^PPI+, FED+^ patients [11]. A more recent report by Iglesias et al. found that out of 9 EoE^PPI+^ patients that trialed FED monotherapy, 6 patients had EoE^PPI+, FED+^ [12]. Our findings are comparable to Iglesias et al.’s, as we found 59.09% of EoE^PPI+^ patients that trialed FED monotherapy were histologically responsive to both PPI monotherapy and FED monotherapy.

Of note, histologic criteria for remission of EoE vary from study to study [13, 14]. In our work, we utilized a more stringent threshold for histologic remission of EoE (≤ 10 eos/hpf vs. < 15 eos/hpf) [11, 12] because we were interested in investigating maintenance therapy for EoE. In addition, although there is little research on the value of lower histologic thresholds [13–15], they have been suggested to be a better predictor of symptomatic and endoscopic response [15]. Since we used peak eosinophil counts as our primary endpoint for remission of EoE, we decided on a more stringent histologic threshold of EoE to account for limitations in our study design.

Adding to previous works, we prospectively followed a cohort of 9 EoE^PPI+, FED+^ patients and 6 EoE^PPI+, FED−^ patients. While EoE^PPI+, FED−^ patients followed a maintenance treatment of PPI monotherapy, EoE^PPI+, FED+^ patients followed maintenance therapy of PPI monotherapy (11.11%; Table 5), FED monotherapy (55.56%, Table 5), or FED with PPI on an as needed basis (33.33%, Table 5). Therefore, trial of FED monotherapy in EoE^PPI+^ patients has the potential to increase options for maintenance therapy. During the observation period, no patients on maintenance therapy had exacerbations of EoE while on a maintenance therapy that warranted urgent EGD or follow up visit (Table 3). More longitudinal research is needed to confirm that maintenance therapy prevents re-activation of EoE long term.

We administered a survey to a cohort of 9 EoE^PPI+, FED+^ patients and 6 EoE^PPI+, FED−^ patients. A majority of patients answered that they underwent this process due to concerns about long term medication usage (60%; Table 4), and that they would recommend this process to another patient with EoE (93.33%; Table 4). A majority of patients agreed that this process helped them identify a treatment plan that aligned with their lifestyle and beliefs (80%; Table 4). While trialing treatment plans for EoE is associated with additional burden [9, 10], our work shows that for patients who are motivated for this process, it can be invaluable to identify more treatment plans that grant the patient flexibility to choose options that are more congruent with the patient’s lifestyle.

Our work has broader implications for the management of EoE. Although a vast majority of current guidelines focus on the importance of identifying a plan that induces remission of EoE [3, 4], our research emphasizes options for patients after histologic remission of EoE is achieved. We suggest that in patients with EoE responsive to a first-line therapy, trial of other first-line monotherapy can be offered to the patient, although further research should be done for other permutations of first-line therapies.

There are several limitations to our study because patients were initially identified retrospectively. We were unable to control for the specific FED that patients underwent. The FED that each patient trialed was selected by patient-clinician shared decision making, but specific reasoning could not be abstracted from the clinical notes. In addition, during Phase 1, our patients did not have a regimented washout period between treatment trials. Therefore, it is plausible that patient’s EoE did not histologically recur after PPI cessation and initiation FED monotherapy. However, a subset of our patients did have histologic recurrence during FED monotherapy trial and EGD was done after at least 8 weeks of treatment, suggesting that EoE would have histologically recurred if the patient was not responsive to FED monotherapy. Furthermore, we were unable to accurately capture metrics commonly used in EoE research, such as validated symptoms outcome measures or endoscopic reference scores (EREFS). Future research investigating FED monotherapy as an alternative treatment for EoE^PPI+^ should standardize the precise FED used, include a washout period between treatments, and include other endpoints utilized in EoE research, like EREFs.

Phase 2 of our study also had various limitations. In our study, healthcare resource utilization was studied by quantifying the number of urgent visits, EGDs, and food impactions. These metrics are not comprehensive of all the healthcare resources utilized by EoE patients. We also cannot preclude that patients were seen at other centers. Additionally, our survey results suffer from sampling bias, as it was only administered to patients who were motivated enough to try FED monotherapy after already knowing that histologic remission of their EoE was induced after PPI monotherapy. Furthermore, our questionnaire is not validated, but can still provide qualitative insights on patient perspectives.

General limitations to our study include our relatively small sample size and that our study was done at a single center located in a major metropolitan area in the United States. These factors may hinder the ability to extrapolate conclusions from our study to other patient populations. Given these large limitations, further work is warranted to clarify if offering FED monotherapy can become the standard of care after remission with PPI treatment.

## Conclusions

We present the largest cohort of EoE^PPI+^ patients that trialed FED monotherapy. Our quantitative findings show that a proportion of EoE^PPI+^ patients (59.09%) are responsive to FED monotherapy as well. By following a prospective cohort of these patients, we did not identify any poor outcomes such as reactivation of EoE. Through our qualitative survey results, we found that this process can identify maintenance treatments for patients' EoE that are congruent for their lifestyle. Our study highlights that clinical management guidelines for EoE should be extended past identifying a single treatment plan that induces remission of EoE.

## Supplementary Information

**Additional file 1: Table S1.** Total patient characteristics from Phase 1. **Table S2.** Prospective cohort characteristics from Phase 2.

## Data Availability

All relevant de-identified data, analytical methods, and study materials are stored in HIPAA-compliant cloud-based storage, and access to these files will be provided on request, by contacting J.L. at drjohnleung@bfac.org.

## Abbreviations

EREFS: Endoscopic reference scores
EoE: Eosinophilic esophagitis
EoE^PPI+^: Eosinophilic esophagitis responsive to PPI monotherapy
EoE^PPI+, FED+^: Eosinophilic esophagitis responsive to PPI monotherapy and FED monotherapy
EoE^PPI+, FED^^−^: Eosinophilic esophagitis responsive to PPI monotherapy but not FED monotherapy
eos/hpf: Eosinophils per high-power field
EGDs: Esophagogastroduodenoscopies
FED: Food elimination diet
4FED: Four-food elimination diet
ICD-10: International classification of diseases, Tenth revision
IQR: Interquartile range
PPI: Proton pump inhibitor
2FED: Two-food elimination diet
