# Supplementary material for: Food elimination diet is a viable alternative therapy for eosinophilic esophagitis responsive to proton pump inhibitors
Source: BMC Gastroenterol. 2023 Mar 9;23:60. doi: 10.1186/s12876-023-02703-9 (PMC9997030; doi:10.1186/s12876-023-02703-9)
Supplement: Supplementary file 1 — Additional file 1: Table S1. Total patient characteristics from Phase 1. Table S2. Prospective cohort characteristics from Phase 2. [file 12876_2023_2703_MOESM1_ESM.docx]

**SUPPLEMENTARY MATERIALS**

**Food elimination diet is a viable alternative therapy for eosinophilic esophagitis responsive to proton pump inhibitors**

Twan Sia, BA^†^, Evan Cunningham, BS^†^, Megan Miller, BS^†^, Rebecca Nitschelm, BS^†^, Riki Tanaka, BS^†^, Taylor Epstein, BS^†^, Kendall Garrett, BS^†^, Amy Huang, BS^†^, Daniel Pak^†^, Ally Scheve, BA^†^, John Leung, MD^†^

^†^Boston Specialists, Boston, MA

Corresponding author:

John Leung, MD

Boston Specialists

65 Harrison Ave Suite #201

Boston, MA 02111

Phone: (617) 804-6767

Fax: (877) 726-8492

[drjohnleung@bfac.org](mailto:drjohnleung@bfac.org)

**This file includes:** Tables S1-2.

**Table S1. Total patient characteristics from Phase 1.**

| **Baseline** | | | | | | **PPI monotherapy** | | | **FED monotherapy** | | |  |  |
| --- | --- | --- | --- | --- | --- | --- | --- | --- | --- | --- | --- | --- | --- |
| **Patient ID** | **Age (years)** | **Sex** | **Atopic conditions** | **Peak eos/hpf** | **Symptoms** | **Treatment plan** | **Peak eos/hpf** | **Symptoms** | **Treatment plan** | **Peak eos/hpf** | **Symptoms** | **Responsiveness** | **Enrolled in Phase 2?** |
| 1 | 26.9 | Female | Allergic rhinitis, food allergy (almonds, goats’ milk) | 87 | Abdominal pain, heartburn, vomiting | Omeprazole 40 mg twice daily | 0 | Vomiting | Dairy, wheat FED | 10 | Heartburn | EoE^PPI+, FED+^ | No |
| 2 | 44 | Male | None | 100 | Dysphagia, heartburn | Omeprazole 40 mg once daily | 0 | Asymptomatic | Dairy FED | 1 | Heartburn | EoE^PPI+, FED+^ | No |
| 3 | 37.9 | Male | None | 50 | Dysphagia | Omeprazole 40 mg once daily | 0 | Asymptomatic | Dairy, wheat FED | 1 | Heartburn | EoE^PPI+, FED+^ | No |
| 4 | 27.5 | Female | Allergic rhinitis, food allergy (shrimp) | 85 | Abdominal pain, dysphagia, heartburn | Omeprazole 40 mg twice daily | 0 | Abdominal pain, heartburn | Dairy, wheat, soy, egg FED | 1 | Asymptomatic | EoE^PPI+, FED+^ | No |
| 5 | 39.5 | Female | Allergic rhinitis | 36 | Dysphagia, vomiting | Omeprazole 40 mg twice daily | 3 | Asymptomatic | Dairy, wheat FED | 60 | Asymptomatic | EoE^PPI+, FED-^ | No |
| 6 | 32.5 | Male | None | 150 | Dysphagia, food impaction | Omeprazole 40 mg once daily | 6 | Asymptomatic | Dairy, wheat, soy, egg, nuts FED | 90 | Dysphagia | EoE^PPI+, FED-^ | No |
| 7 | 34.8 | Female | Allergic rhinitis, asthma, food allergy (peanuts and tree nuts) | 80 | Dysphagia | Omeprazole 40 mg twice daily | 0 | Dysphagia | Dairy, wheat FED | 30 | Asymptomatic | EoE^PPI+, FED-^ | No |
| 8 | 27.6 | Female | None | 100 | Dysphagia | Omeprazole 40 mg once daily | 0 | Asymptomatic | Dairy, wheat FED | 1 | Dysphagia | EoE^PPI+, FED+^ | Yes |
| 9 | 38.4 | Male | Food allergy (stone fruits) | 45 | Chest pain | Omeprazole 40 mg twice daily | 6 | Asymptomatic | Dairy, wheat FED | 0 | Asymptomatic | EoE^PPI+, FED+^ | Yes |
| 10 | 29.5 | Male | Food allergy (eggs) | 20 | Dysphagia | Omeprazole 40 mg once daily | 10 | Asymptomatic | Dairy, wheat FED | 1 | Asymptomatic | EoE^PPI+, FED+^ | Yes |
| 11 | 35.1 | Male | Allergic rhinitis | 20 | Dysphagia, heartburn | Omeprazole 40 mg once daily | 0 | Dysphagia, heartburn | Dairy FED | 10 | Asymptomatic | EoE^PPI+, FED+^ | Yes |
| 12 | 59.1 | Male | None | 30 | Dysphagia, heartburn | Omeprazole 40 mg twice daily | 4 | Dysphagia | Dairy, wheat FED | 6 | Dysphagia, heartburn | EoE^PPI+, FED+^ | Yes |
| 13 | 52.8 | Female | None | 25 | Chest pain, dysphagia | Omeprazole 20 mg twice daily | 10 | Asymptomatic | Dairy FED | 10 | Asymptomatic | EoE^PPI+, FED+^ | Yes |
| 14 | 31.3 | Male | None | 38 | Dysphagia | Omeprazole 40 mg twice daily | 1 | Heartburn | Dairy, wheat FED | 6 | Asymptomatic | EoE^PPI+, FED+^ | Yes |
| 15 | 58.9 | Male | None | 20 | Dysphagia, food impaction, heartburn | Omeprazole 40 mg twice daily | 4 | Asymptomatic | Dairy FED | 10 | Asymptomatic | EoE^PPI+, FED+^ | Yes |
| 16 | 39.8 | Female | Allergic rhinitis, asthma | 20 | Dysphagia, heartburn | Omeprazole 40 mg twice daily | 2 | Asymptomatic | Dairy, wheat FED | 9 | Asymptomatic | EoE^PPI+, FED+^ | Yes |
| 17 | 32.5 | Male | None | 75 | Chest pain, heartburn | Omeprazole 40 mg twice daily | 10 | Asymptomatic | Dairy, wheat FED | 70 | Asymptomatic | EoE^PPI+, FED-^ | Yes |
| 18 | 29.1 | Female | Allergic rhinitis, asthma | 33 | Chest pain | Omeprazole 40 mg twice daily | 3 | Asymptomatic | Dairy, wheat FED | 15 | Asymptomatic | EoE^PPI+, FED-^ | Yes |
| 19 | 28.3 | Male | None | 54 | Abdominal pain, dysphagia, regurgitation | Omeprazole 40 mg twice daily | 6 | Dysphagia, regurgitation | Dairy, wheat, soy FED | 27 | Asymptomatic | EoE^PPI+, FED-^ | Yes |
| 20 | 55 | Male | None | 15 | Dysphagia | Omeprazole 40 mg twice daily | 6 | Asymptomatic | Dairy, wheat FED | 30 | Asymptomatic | EoE^PPI+, FED-^ | Yes |
| 21 | 25 | Male | Food allergy (shellfish) | 89 | Dysphagia, heartburn | Omeprazole 40 mg twice daily | 2 | Asymptomatic | Dairy, wheat FED | 30 | Dysphagia, heartburn | EoE^PPI+, FED-^ | Yes |
| 22 | 33.3 | Female | Allergic rhinitis | 80 | Dysphagia, food impaction | Omeprazole 40 mg once daily | 2 | Dysphagia | Dairy, wheat FED | 37 | Asymptomatic | EoE^PPI+, FED-^ | Yes |

EoE^PPI+, FED+^, eosinophilic esophagitis responsive to proton pump inhibitor monotherapy and food elimination diet monotherapy; EoE^PPI+, FED-^, eosinophilic esophagitis responsive to proton pump inhibitor monotherapy and food elimination diet monotherapy; eos/hpf, eosinophils per high-power field; FED, food elimination diet; PPI, proton pump inhibitors.

**Table S2. Prospective cohort characteristics from Phase 2.**

| **Patient ID** | **Follow up duration (years)** | **Food impactions requiring urgent EGD** | **Reported recurrence of EoE symptoms** | **Repeat EGD for histologic monitoring of EoE** | **Identified histologic reactivation of EoE** | **Repeat EGD to trial other treatment plans** |
| --- | --- | --- | --- | --- | --- | --- |
| 8 | 0.1 | 0 | 0 | 0 |  | 0 |
| 9 | 1.1 | 0 | 0 | 1 | No | 0 |
| 10 | 1.5 | 0 | 0 | 0 |  | 1 |
| 11 | 1.5 | 0 | 0 | 0 |  | 0 |
| 12 | 2.2 | 0 | 0 | 0 |  | 0 |
| 13 | 2.4 | 0 | 0 | 0 |  | 1 |
| 14 | 2.5 | 0 | 0 | 0 |  | 3 |
| 15 | 2.5 | 0 | 0 | 0 |  | 0 |
| 16 | 2.7 | 0 | 0 | 1 | No | 2 |
| 17 | 0.1 | 0 | 0 | 0 |  | 1 |
| 18 | 0.6 | 0 | 0 | 0 |  | 2 |
| 19 | 1.1 | 0 | 0 | 1 | No | 1 |
| 20 | 1.1 | 0 | 0 | 0 |  | 1 |
| 21 | 2.8 | 0 | 0 | 0 |  | 0 |
| 22 | 2.9 | 0 | 0 | 0 |  | 0 |

EGD, esophagoduodenoscopy; EoE, eosinophilic esophagitis.
